# Low Cholesterol Concentrations and the Risk of Peptic Ulcer Bleeding: A Retrospective Cohort Study

**DOI:** 10.3390/jcm14124056

**Published:** 2025-06-08

**Authors:** Mehmet Kök, Süleyman Dolu, Mehmet Emin Arayici, Gökhan Köker, Lütfullah Zahit Koç, Ayhan Hilmi Cekin

**Affiliations:** 1 Department of Internal Medicine, University of Health Sciences, Antalya Training and Research Hospital, Antalya 07100, Turkey; gokhan.koke@sbu.edu.tr (G.K.); lutfullahzahit.koc@sbu.edu.tr (L.Z.K.); 2 Department of Gastroenterology, Faculty of Medicine, Dokuz Eylül University, Izmir 35340, Turkey; suleyman.dolu@deu.edu.tr; 3 Department of Biostatistics and Medical Informatics, Faculty of Medicine, Dokuz Eylül University, Izmir 35340, Turkey; mehmet.e.arayici@gmail.com; 4 Department of Gastroenterology, University of Health Sciences, Antalya Training and Research Hospital, Antalya 07100, Turkey

**Keywords:** low cholesterol, upper gastrointestinal bleeding, bleeding peptic ulcer, risk assessment

## Abstract

**Background:** Hypolipidemia has been shown to be a factor that elevates the risk of bleeding. This study aimed to investigate the relationship between cholesterol levels and the risk of peptic ulcer bleeding (PUB). **Methods**: A total of 15,547 patients who underwent esophagogastroduodenoscopy (EGD) between May 2017 and December 2020 were screened. Of these, 317 were included in this retrospective cohort study. PUB was diagnosed by EGD. Serum cholesterol levels were measured at six-month intervals, and cumulative mean cholesterol concentrations were calculated using all available data during the observation period. **Results**: Ulcer bleeding was detected in 173 (45.6%) patients with peptic ulcer. Patients with bleeding peptic ulcer exhibited significantly lower TC, LDL-C, and HDL-C levels compared to non-bleeding peptic ulcer patients and controls (*p* < 0.001). The optimal LDL cut-off was 91.50 (*p* < 0.001), with 75.1% sensitivity and 24.1% specificity. For HDL, the cut-off was 42.50 (*p* < 0.001), yielding 72.3% sensitivity and 30.1% specificity. A one-unit increase in LDL and HDL reduced the risk of PUB by 0.95-fold and 0.97-fold, respectively. **Conclusions**: Our findings suggest that reduced cholesterol levels, particularly LDL-C and HDL-C, may elevate the risk of PUB. Therefore, monitoring cholesterol levels in peptic ulcer patients, especially those at higher bleeding risk, could be beneficial.

## 1. Introduction

Upper gastrointestinal bleeding (UGIB) is defined as bleeding occurring proximal to the ligament of Treitz. Recent studies have indicated a reduction in mortality, likely attributable to the introduction of proton pump inhibitors (PPI), improved detection and treatment of Helicobacter pylori (H. pylori), and advancements in endoscopic and interventional radiologic techniques [1,2]. Peptic ulcer (PU) remains the most common cause despite declining incidence [3]. Available data indicate that the incidence of UGIB is approximately 100 cases per 100,000 individuals per year, with a mortality rate generally ranging between 6 and 10% [4]. Furthermore, healthcare utilization and expenditures for patients with UGIB increased for at least 12 months following the event [5]. The most common risk factors for UGIB include H. pylori infection, the use of nonsteroidal anti-inflammatory drugs (NSAIDs), aspirin, other anti-platelet and anti-coagulant medications, along with comorbidities and older age [6,7].

The introduction of proprotein convertase subtilisin/kexin type 9 (PCSK9) inhibitors, which enable a reduction in low-density lipoprotein cholesterol (LDL-C) to levels previously unattainable with statin therapy, has established the principle of “the lower, the better” as the prevailing approach for LDL-C lowering in the prevention of atherosclerotic cardiovascular disease (ASCVD) [8]. However, observational research from population-based cohorts has indicated a U-shaped relationship between baseline LDL-C concentrations and all-cause mortality [9]. Over the past two decades, studies involving East Asian populations and Caucasian women have demonstrated a link between low levels of LDL-C and an increased risk of hemorrhagic stroke in individuals from the general population who are not receiving antithrombotic treatment [10,11,12]. Furthermore, growing evidence has indicated that reduced levels of low-density LDL-C are linked to an increased risk of bleeding in patients receiving dual antiplatelet therapy (DAPT) after undergoing percutaneous coronary intervention (PCI) [13,14,15,16]. The precise mechanisms responsible for the increased bleeding risk associated with low cholesterol remain unclear. With regard to the risk of haemorrhagic stroke, it has been hypothesized that maintaining sufficient lipid levels is vital for preserving the structural integrity and fluidity of cellular membranes. Consequently, low cholesterol levels may contribute to endothelial cell fragility and increased permeability of the blood–brain barrier, potentially playing a role in the pathogenesis of haemorrhagic stroke associated with low cholesterol exposure [17,18]. Recent findings indicate that cholesterol may have a significant role in regulating platelet count and function. For instance, reduced levels of LDL-C have been associated with diminished platelet aggregation, impaired thrombopoiesis, and a subsequent reduction in platelet count.

Gastrointestinal bleeding represents the most common haemorrhagic complication in patients receiving DAPT after PCI, particularly among those with a prior history of peptic ulcers [19]. Notably, a recent investigation assessing potential long-term on-target effects of PCSK9 inhibitors explored the relationship between an LDL-C-lowering variant in the PCSK9 gene and clinical outcomes in the UK Biobank, revealing a previously unrecognized association between this variant and an elevated risk of peptic ulcer disease [20]. Since the gastrointestinal epithelium undergoes continuous renewal driven by stem cells to counteract mechanical injury from luminal contents, reduced cholesterol levels may impair epithelial proliferation within the gastrointestinal tract, potentially contributing to delayed ulcer healing [21]. Considering that intensive lipid-lowering and high-potency antithrombotic treatments represent the current standard for secondary prevention of ASCVD, these observations may carry significant clinical relevance; however, existing research on this matter remains limited. Given these considerations, our study sought to more thoroughly examine the association between low serum cholesterol levels and the risk of PU bleeding.

## 2. Materials and Methods

### 2.1. Study Design and Patient Selection

**Figure 1:**
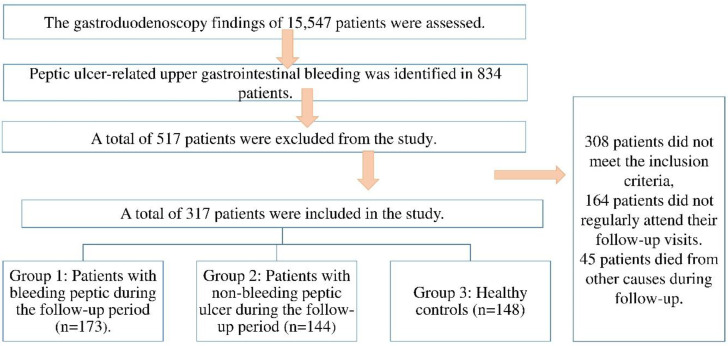
Subject Selection.

In this retrospective cohort study, the esophagogastroduodenoscopy (EGD) findings of 15,547 patients who underwent EGD between May 2017 and December 2020 at our tertiary care center were reviewed. Among these, 834 patients were diagnosed with peptic ulcer-related upper gastrointestinal bleeding. After applying the predefined inclusion and exclusion criteria, a total of 517 patients were excluded due to factors such as incomplete data, history of malignancy, use of lipid-lowering agents during the follow-up period, or lack of follow-up cholesterol measurements. The final study population consisted of three groups. Group 1 included 173 patients who were diagnosed with peptic ulcer bleeding (PUB) during the follow-up period based on endoscopic confirmation. Group 2 comprised 144 patients with peptic ulcers who did not experience bleeding during the same follow-up period. Group 3 (control group) consisted of 148 healthy individuals without any history of peptic ulcer or gastrointestinal bleeding, who were matched by age and sex and met the same inclusion criteria. These individuals were followed over the same time frame as the patient groups. A flow diagram summarizing patient selection and group classification is provided in Figure 1.

In our clinic, patients with PU undergo monthly follow-ups for the first three months after diagnosis, followed by semi-annual check-ups, with control esophagogastroduodenoscopy (EGD) scheduled 8 to 12 weeks post-treatment. Patients diagnosed with PU between May 2017 and December 2020, who had been regularly followed for at least three years, or who experienced PU bleeding following a follow-up EGD, were included in the study. Data on age, gender, smoking status, use of anti-aggregant, anti-coagulant, NSAIDs, and lipid-lowering medications (e.g., statins), concomitant comorbidities affecting the coagulation system such as malignancy, chronic liver and kidney failure, and previous history of UGIB were obtained from the patient information registry system of our clinic. The classification of PU was described using the Forrest classification on EGD [13]. The inclusion criteria for this study are as follows: (1) Patients aged 18 years and older; (2) Patients diagnosed with PU confirmed through EGD and biopsy, with at least one biopsy taken from each of the four quadrants of the ulcer in our clinic; (3) Patients with endoscopic evidence of bleeding PU in our clinic; (4) Patients who have completed both endoscopic and medical treatment for PU and are asymptomatic; (5) Patients whose follow-up EGD (approximately 8 weeks after treatment) revealed healed or healing ulcers; (5) Patients who were confirmed negative for H. pylori after eradication therapy if H.pylori was detected at the time of diagnosis, as evidenced by negative H. pylori antigen in follow-up biopsy and stool tests. The exclusion criteria are as follows: (1) Participants with incomplete information in their medical history (medication use, comorbidities, history of UGIB, etc.) as previously detailed above; (2) Patients who missed their last two check-ups; (3) Individuals diagnosed with a malignant ulcer; (4) Individuals with solid or hematological malignancies; (5) Individuals with known hemoglobinopathies, coagulopathies or bleeding diatheses (e.g., sickle cell anemia, thalassemia, thrombocytopenia, hemophilia); (6) Individuals with liver dysfunction (e.g., liver cirrhosis) or kidney dysfunction (e.g., chronic renal failure); (7) Participants who began or discontinued the use of lipid-lowering medications after being enrolled in the study.

The control group was selected via a database query from the clinical data registry of healthy volunteers who were admitted to Antalya Training and Research Hospital during the same study period. These individuals had undergone upper gastrointestinal endoscopy (EGD) and relevant laboratory assessments as part of a prospective observational study protocol. They exhibited normal endoscopic findings and met the same inclusion and exclusion criteria as the patient groups. All individuals in the control group received comprehensive information regarding the nature and objectives of the procedures prior to enrollment in the study and throughout the follow-up period. Written informed consent was obtained from each participant before their inclusion. All three groups were monitored over the same follow-up period, enabling the evaluation of lipid parameters (TC, LDL-C, HDL-C, TG) and their association with PUB risk.

### 2.2. Laboratory Parameters

According to our clinic’s follow-up protocol, venous blood samples are collected from all patients in the morning after fasting overnight at each visit. Blood counts, coagulation (prothrombin time (PT), activated partial thromboplastin time (APTT)) and routine biochemical parameters (blood urea nitrogen (BUN), creatinine, alanine transaminase (ALT), and aspartate transaminase (AST), etc.) are evaluated at every visit, and lipid parameters (total cholesterol (TC), triglycerides (TG), HDL-cholesterol (HDL-C), LDL-cholesterol (LDL-C) are checked every six months. The same protocol was used for the participants in the control group.

Blood was collected by using Vacuette^®^ Standard tube holder and Vacuette^®^ 21-gauge, 0.80 × 38 mm multisample needle (Vacuette, Greiner Bio-One, Kremsmünster, Austria). Vacutainer tubes without gel were used for the routine biochemical parameters and lipid parameters were determined by using commercially available assay kits (Abbott) with an autoanalyzer (Architect^®^ c16000, Abbott Diagnostics Division, Abbott Laboratories, Abbott Park, Illinois, United States). The LDL cholesterol concentrations were calculated using the Friedewald formula. Internal and external quality control results for all tests were within the acceptable limits during the study. All samples were separated by centrifugation at 1500× *g* for 15 min and all parameters were measured within 30 min. Hemogram (whole blood count) was analyzed on Sysmex XT-2000i (Sysmex, Kobe, Japan). Trisodium citrated 1/9 tubes were used to obtain plasma samples for coagulation parameters such as PT and APTT. These markers were measured with original reagents on the coagulation analyzer (ACL Futura Plus, Instrumentation Laboratory—IL, Milan, Italy).

Cumulative mean cholesterol concentrations were derived by aggregating all available cholesterol data over the follow-up period. Hypocholesterolemia has been identified in numerous studies as a prevalent characteristic of acute medical conditions, including UGIB and sepsis [4,22]. Therefore, cholesterol levels were not evaluated in such medical conditions.

### 2.3. Statistical Analysis

In the first stage of this study, descriptive analysis of descriptive characteristics (such as age, gender, and medical history) and various laboratory values obtained from groups consisting of individuals with bleeding PU, individuals with non-bleeding PU and normal individuals were performed. In the next stage, the mean values of the various laboratory parameters of the groups were compared. To capture individuals’ long-term cholesterol trends, we determined the current cumulative mean cholesterol concentrations using all available cholesterol measurements obtained during the follow-up period or until the diagnosis of UGIB. Before proceeding to the average comparison tests of the groups, the distribution structures were examined. Skewness and kurtosis of the group mean skewness and kurtosis Z-tests, and Kolmogorov–Smirnov/Shapiro–Wilk tests were conducted to determine the distribution structure. One-way analysis of variance was used due to the normal distribution of all score distributions and the presence of more than two groups. In the next stage of the research, regression analysis was performed to determine the correct classification percentage of the groups and to create a model for classification. Multinomial logistic regression analysis was used as the dependent variable was categorical and the number of groups was three. In the final stage of the research, the cutoff points of the independent variables contributing to the regression model were determined using the ROC curve.

## 3. Results

As previously mentioned, the participants were categorized into three groups: the bleeding PU group, the non-bleeding PU group and the control group. Seventy-nine of 173 patients (45.6%) in the bleeding PU group and 66 of 144 (45.8%) patients in the GC group were female. The median ages of the groups were 57.18 ± 18.38 and 60.19 ± 13.19, respectively, in the bleeding PU group and the non-bleeding PU group. There was no significant difference between the bleeding PU and non-bleeding PU group in terms of age and sex distribution. The control group consisted of 148 age and sex-matched healthy participants. No statistically significant difference was observed between the patient groups concerning predisposing medication, statin and smoking use (Table 1).

Upon comparing the laboratory values of the participants, we found that hemoglobin levels were lower and that neutrophil counts were higher in the bleeding PU group compared to the other groups (*p* < 0.001 for both). No statistically significant differences were identified in the other hemogram parameters between the groups. The analysis of coagulation parameters was not conducted because some patients in the PU groups were using anticoagulants with different mechanisms of action. When biochemical tests were evaluated, no significant difference was found between the groups in terms of AST, ALT, BUN, and creatinine values. Upon analyzing lipid parameters, it was found that LDL-C, HDL-C, and TC levels were statistically significantly lower in the bleeding PU group compared to the other groups (*p* < 0.001 for all). The effect size coefficient for the observed significant differences was found to indicate a medium effect for neutrophils (η^2^ = 0.10) and a large effect size for hemoglobin (η^2^ = 0.12), LDL-C (η^2^ = 0.33), HDL-C (η^2^ = 0.23), and total cholesterol (η^2^ = 0.34) (Table 2).

The results of the Receiver Operating Characteristic (ROC) curve analysis performed to evaluate the diagnostic performance of LDL and HDL risk factors in patients with bleeding PU are presented in Table 3. The AUC value for the LDL risk factor was 0.843 (95% CI: 0.786–0.900), indicating the high diagnostic performance of LDL in PU bleeding. Based on the analysis, the optimal cut-off point for LDL was determined to be 91.50 (*p* < 0.001). The calculated sensitivity for the LDL risk factor was 75.1%, while the specificity was 24.1%. For the HDL risk factor, the AUC value was 0.785 (95% CI: 0.723–0.846), also demonstrating strong diagnostic performance in identifying PU bleeding. The optimal cut-off point for HDL was identified as 42.50 (*p* < 0.001), with a sensitivity of 72.3% and a specificity of 30.1% (Table 3). The associations between LDL and HDL values of patients with bleeding PU and the severity of PU bleeding were analyzed using the point biserial correlation coefficient. The results indicated that the relationships between the degree of bleeding and both LDL-C (r = 0.07, *p* = 0.443) and HDL-C (r = 0.04, *p* = 0.688) were statistically insignificant and of small magnitude.

A multinomial logistic regression analysis was conducted with disease subgroups as the dependent variable, and medication use, smoking status, age, and lipid parameters as the independent variables. Following the analysis, variables such as age (*p* = 0.1306), smoking status (*p* = 0.862), medication use (*p* = 0.109), total cholesterol (*p* = 0.940), and triglycerides (*p* = 0.760) were excluded from further consideration due to their non-significant predictive coefficients. We found significant predictive coefficients for both LDL-C and HDL-C (*p* < 0.001 for both). The results of the model and variable compliance values of the multinomial logistic regression analysis with LDL-C and HDL-C as independent variables are provided in Table 4.

The coefficients of the multinomial logistic regression model are shown in Table 5. Upon examination, it is evident that the dependent variable consists of three categories, resulting in two sets of logistic regression coefficients. The first set represents the comparison of the reference category, “normal individuals”, with the category “patients with bleeding PU.” The second set of coefficients compares the “patients with non-bleeding PU” category to the reference category. Upon reviewing the analysis results, it is observed that the estimated slope coefficient (B) for the LDL variable is −0.05, with an odds ratio of 0.95. This indicates that, after controlling for other predictor variables, a one-unit increase in LDL results in a 0.95-fold reduction in the risk of PU bleeding. Similarly, the slope coefficient for the HDL variable is −0.04, with an odds ratio of 0.97. This suggests that, after adjusting for other predictors, a one-unit increase in HDL decreases the likelihood of PU bleeding by 0.97 times.

## 4. Discussion

In the present study, we observed that patients with bleeding PU had lower TC, LDL-C, and HDL-C levels compared to both non-bleeding PU patients and the control group. We also found that this effect persisted after adjusting for other predictor variables and that a one-unit increase in LDL reduced the risk of PU bleeding by 0.95-fold, while a one-unit increase in HDL reduced the probability of PU bleeding by 0.97-fold.

UGIB presents a significant clinical challenge and remains a major cause of morbidity and mortality [7]. Given these figures, it is essential to identify modifiable risk factors. Besides established risks such as smoking and the use of certain medications, low cholesterol levels may also contribute as a potential risk factor for UGIB. Several epidemiologic studies have reported an inverse association between LDL-C concentrations and ICH risk. Over the past two decades, numerous epidemiological studies have documented an inverse relationship between LDL-C levels and the risk of ICH [13,23,24]. For instance, Wang et al. conducted a comprehensive meta-analysis that demonstrated an inverse relationship between lower TC levels and the risk of hemorrhagic stroke [23]. Low LDL-C levels can influence endothelial stability and function through multiple inflammatory and oxidative stress pathways. For example, oxidized LDL (oxLDL), which is derived from LDL particles, plays a central role in endothelial cell injury by reducing nitric oxide levels, increasing reactive oxygen species (ROS) production, and activating NF-κB, leading to enhanced secretion of proinflammatory cytokines like IL-8. These changes promote endothelial dysfunction (ED), which is a known precursor to increased vascular fragility and permeability. Additionally, dysregulation in endothelial energy metabolism, immune activation, and barrier dysfunction are also implicated in vascular injury. While our study did not directly examine these molecular mechanisms, our findings align with these pathophysiological insights and support the need for future investigations that integrate clinical and mechanistic approaches [25]. Additionally, many studies have shown that low TG levels are associated with an increased risk of ICH. However, findings from prospective cohort studies indicate that the summary relative risk of hemorrhagic stroke for each 1 mmol/L increase in HDL-C, based on a dose–response analysis, is 1.17 (95% CI 1.02–1.35). This suggests that elevated HDL-C levels may be linked to an increased risk of ICH. In summary, the majority of studies indicate that lower TC, LDL-C, and TG levels, combined with higher HDL-C levels, are associated with an elevated risk of ICH. Moreover, several studies have identified a correlation between hypercholesterolemia and a decreased risk of bleeding in patients undergoing antiplatelet therapy. For instance, Lin et al. showed that hypolipidemia (LDL-C < 130 mg/dL) increased the risk of developing ICH in patients with acute ischemic stroke treated with IV thrombolysis [24]. Yang et al. demonstrated that reduced LDL-C levels elevate the risk of in-hospital bleeding in patients undergoing percutaneous coronary intervention (PCI) for acute coronary syndromes [13]. In this current study, we observed that PU patients with bleeding exhibited lower levels of TC, and LDL-C compared to patients with non-bleeding PU and the control group, consistent with the literature. However, HDL-C levels were lower in the bleeding PU group than in the other groups, and there was no significant difference in TG levels between the groups. Moreover, these effects remained significant after adjusting for other predictor variables. Specifically, a one-unit increase in LDL was associated with a 0.95-fold reduction in the risk of PU bleeding, while a one-unit increase in HDL decreased the likelihood of PU bleeding by 0.97-fold. Although our study demonstrated a significant association between low cholesterol levels and increased bleeding risk, a 3-year follow-up period may be insufficient to fully assess long-term cholesterol dynamics or capture delayed bleeding events. Furthermore, while our findings are consistent with previous literature suggesting that hypocholesterolemia may predispose to hemorrhagic events, the potential cumulative effects of chronic low cholesterol—particularly on gastrointestinal mucosal integrity—were not directly evaluated.

Most studies examining the relationship between ICH and cholesterol levels have utilized a single baseline measurement of LDL-C. However, cholesterol levels can fluctuate over time due to natural variations, lipid-lowering treatments, and acute stress conditions such as sepsis or trauma [26,27]. For instance, Hrabovský et al. measured cholesterol levels in patients with UGB on the first, third, and sixth days of hospitalization, finding that the lowest levels occurred upon admission, followed by a gradual increase [4]. In the current study, participants’ cholesterol levels were measured twice at six-month intervals. However, this approach may not fully capture short-term fluctuations due to acute stressors such as infections, inflammation, or other transient physiological changes. This is an acknowledged limitation, as lipid levels can vary dynamically in response to systemic stress. To minimize potential confounding effects, we excluded participants who initiated or discontinued lipid-lowering therapy during the study period. By doing so, we aimed to ensure more stable baseline lipid profiles.

Our study has several limitations. First, the retrospective design may have introduced selection bias. Second, although the initial cohort included 834 patients with peptic ulcer, only 317 were eligible for inclusion due to incomplete follow-up or missing clinical data. This necessary exclusion reduced the final sample size, which may limit statistical power and generalizability. However, rigorous selection ensured data accuracy and minimized confounding. Third, the follow-up period was relatively short, only three years, which may not fully capture long-term outcomes. Therefore, more comprehensive prospective research is warranted to further investigate this issue. LDL cholesterol levels were estimated using the Friedewald formula, which can be affected by certain metabolic conditions, such as hypertriglyceridemia. We did not directly measure serum LDL-C levels. Additionally, some confounders—like diet, genetics, and inflammation—could not be assessed due to data limitations. While statin use was recorded, dosage and duration details were unavailable, limiting its interpretability.

In conclusion, this cohort study demonstrated a significant association between low serum cholesterol levels—particularly LDL-C and HDL-C—and an increased risk of PUB. Patients with bleeding ulcers exhibited lower levels of total cholesterol, LDL-C, and HDL-C compared to both non-bleeding ulcer patients and healthy controls, and these associations remained significant after adjusting for potential confounding variables. ROC and multinomial logistic regression analyses further supported the predictive value of LDL-C and HDL-C in identifying patients at risk for bleeding. These findings suggest that serum cholesterol levels, which are routinely measured in clinical practice, may serve as useful indicators for bleeding risk stratification in patients with peptic ulcer disease. Nevertheless, further large-scale prospective studies are needed to confirm these findings and explore the underlying mechanisms.

## Figures and Tables

**Table 1:** Sociodemographic features of groups.

	Subgroup	Group 1		Group 2		Group 3		*p* Value
		n	%	n	%	n	%	
Gender	Woman	79	45.6	66	45.8	68	45.9	0.22
	Male	94	54.3	78	54.2	80	54.1	
Age ($\bar{X}\pm$ SD)		57.18 ± 18.38		60.19 ± 13.19		54.40 ± 15.08		0.314
Statin use	Yes	23	13.2	29	20.1	28	18.9	
	None	150	76.8	115	79.9	120	81.1	0.075
NSAID use	Yes	44	25.4	38	26.3	34	22.9	
	None	129	74.6	105	38.4	114	77.1	0.345
Anti-aggregant use	Yes	57	32.9	36	33.3	45	30.4	
	None	116	67.1	108	66.7	103	69.6	0.654
Anti-coagulant use	Yes	19	10.9	16	11.1	13	8.7	0.443
	None	156	89.1	128	88.9	135	91.3	
Smoking	Yes	48	27.4	38	26.3	35	23.6	
	None	125	77.6	106	73.7	113	76.4	0.38

Abbreviations: NSAID = nonsteroidal anti-inflammatory drug. Group 1: Patients with bleeding peptic ulcer; Group 2: Patients with non-bleeding peptic ulcer; Group 3: control.

**Table 2:** Laboratory values of the participants.

Laboratory Parameters	Group	N	Mean ± SD	*p*	Difference	η^2^
Hemoglobin	1	173	9.56 ± 2.20	<0.001^ †^	2-1 3-1	
	2	144	15.66 ± 16.16			0.12
	3	148	13.35 ± 1.97			
Thrombocyte	1	173	221.63 ± 19.65	0.064		
	2	144	265.72 ± 23.35			
	3	148	275.26 ± 22.16			
Neutrophil	1	173	6.40 ± 3.22	<0.001^ †^	1-2 1-3	
	2	144	4.74 ± 2.23			0.10
	3	148	4.22 ± 1.24			
Lymphocyte	1	173	2.28 ± 3.67	0.779	-	
	2	36	2.71 ± 3.71			
	3	47	2.33 ± 0.78			
ALT	1	173	24.03 ± 4.16	0.42		
	2	144	26.08 ± 3.26			
	3	148	28.83 ± 4.27			
AST	1	173	26.03 ± 4.16	0.36		
	2	144	25.08 ± 3.26			
	3	148	28.83 ± 4.27			
BUN	1	173	12.34 ± 3.16	0.24		
	2	144	13.82 ± 3.46			
	3	148	12.74 ± 4.25			
Creatinine	1	173	0.88 ± 0.16	0.42		
	2	144	0.83 ± 0.26			
	3	148	0.89 ± 0.27			
LDL-C	1	173	73.79 ± 23.80	<0.001 ^†^	2-1 3-1	
	2	144	110.36 ± 37.06			0.33
	3	148	121.38 ± 37.70			
HDL-C	1	173	37.02 ± 11.51	<0.001^ †^	2-1 3-1	0.23
	2	144	54.04 ± 17.33			
	3	148	53.83 ± 19.80			
Triglyceride	1	173	120.89± 59.84	0.532	-	
	2	144	125.11 ± 68.96			
	3	148	132.13 ± 61.01			
Total Cholesterol	1	173	134.55 ± 30.06	<0.001^ †^	2-1 3-1	
	2	144	185.83 ± 48.58			0.34
	3	148	198.00 ± 51.67			

Abbreviations: ALT = alanine transaminase; AST = aspartate transaminase; BUN = blood urea nitrogen; LDL-C = low-density lipoprotein cholesterol; HDL-C = high-density lipoprotein cholesterol; TG = triglycerides, TC = total cholesterol. Group 1: Patients with bleeding peptic ulcer; Group 2: Patients with non-bleeding peptic ulcer; Group 3: control. ^†^ Statistically significant.

**Table 3:** Diagnostic performance of LDL-C and HDL-C risk factors in patients with peptic ulcer bleeding.

Risk Factor	AUC (95%)	Cut Off	*p*	Sensitivity (%)	Specifity (%)
LDL-C	0.843 (0.786–0.900)	91.50	<0.001	75.1	24.1
HDL-C	0.785 (0.723–0.846)	42.50	<0.001	72.3	30.1

Abbreviations: LDL-C = low-density lipoprotein cholesterol; HDL-C = high -density lipoprotein cholesterol; AUC = Area under curve.

**Table 4:** Values of the Multinomial Logistic Regression Model.

Effect	Probability Ratio Test			Pearson Goodness of Fit Test		Pseudo R^2^	
	−2 log Likelihood	χ2	*p*	χ2	*p*	Cox and Snell	Nagelkerke
Intersection	492.31	174.15	<0.001	673.98	<0.001	0.36	0.44
LDL--C	367.66	49.49	<0.001				
HDL-C	329.08	10.92	0.004				

Abbreviations: LDL-C = low-density lipoprotein cholesterol; HDL-C = high-density lipoprotein cholesterol. Multivariable HR (95% CI) was adjusted for age, medication use, and smoking status.

**Table 5:** Coefficients of the Multinomial Logistic Regression Model.

Group *	Variable	B	SE	Wald	*p*	Exp (B)
1	Intersection	7.77	0.97	63.59	<0.001	
	LDL-C	-0.05	0.01	31.61	<0.001	0.95
	HDL-C	-0.04	0.02	5.84	0.016	0.97
2	Intersection	0.57	0.89	0.41	0.525	
	LDL--C	-0.01	0.01	2.42	0.120	0.99
	HDL-C	0.01	0.01	0.54	0.463	1.01

Abbreviations: LDL-C = low-density lipoprotein cholesterol; HDL-C = high-density lipoprotein cholesterol B = Slope coefficient; SE = Standard error; Exp (β) = Odds ratio. * Reference category is “Control group”. Group 1: Patients with bleeding peptic ulcer; Group 2: Patients with non-bleeding peptic ulcer.

## Data Availability

The data supporting the findings of this study are available from the corresponding author upon reasonable request. Proposals for access should be sent to dr.mehmetkok@hotmail.com.

## Abbreviations

ALT	alanine transaminase
APTT	activated partial thromboplastin time
ASCVD	atherosclerotic cardiovascular disease
BUN	blood urea nitrogen
DAPT	dual antiplatelet therapy
EGD	esophagogastroduodenoscopy
H. pylori	Helicobacter pylori
HDL-C	HDL cholesterol
ICH	intracerebral hemorrhage
LDL-C	LDL cholesterol
PCI	percutaneous coronary intervention
PCSK9	proprotein convertase subtilisin/kexin type 9
PPI	proton pump inhibitors
PT	prothrombin time
ROC	receiver operating characteristic
TC	total cholesterol
TG	triglycerides
UGIB	upper gastrointestinal bleeding
